# Clinical Lectures on Medicine

**Published:** 1871-04

**Authors:** Charles Murchison

**Affiliations:** Physician to the Hospital, and Lecturer on the Practice of Medicine: Consulting Physician and Vice-President of the London Fever Hospital


### CLINICAL LECTURES ON MEDICINE.

Delivered at the Medical Hospital.

BY CHARLES MURCHISON, M. D., LL.D, F. R. S.,

Physician to the Hospital, and Lecturer on the Practice of Medicine: Consulting Physician and Vice-President of the London Fever Hospital.

### LECTURE IV.

### 1. *Case of Rötheln, or German Measels.*

Gentlemen:—The case of Elizabeth R——, who was discharged from the hospital a few days ago, is one that deserves your careful attention.

Elizabeth R——, aged fifteen, was admitted into the Middlesex Hospital on April 14th, 1870. She was house-maid in a gentleman's family. She had had an attack of measles at the age of four, and of scarlet fever at the age of six. On the morning of April 12th she had been quite well, and in the evening she had first complained of headache, loss of appetite, chilliness, and running from the eyes. During the following night she had been very restless and thirsty, and on the morning of the 13th she noticed an eruption on the face, which soon extended over the whole body. The throat from the first had been slightly sore, but there had been no sneeqing nor cough.

On admission, the girl did not look very ill, but her face, chest, arms, legs, and entire body were covered with an eruption consisting of irriegular patches, at many places running into one another so as to form a large red space, but at others quite isolated. The whole eruption presented very much the appearance of measles, but the patches were less crescentic in outline, and had nowhere a decidedly popular character.—Skin dry; temperature 101; pulse 132. The patient sneezed several times within two or three hours of admission, and had a slight running from the nose, and a watery discharge from the eyes, but the conjunctivæ were not injected, and there was no cough nor bronchitic rales in the chest. The tongue was moist, coated with a thin white fur, and red at the edges, but there was no marked enlargment of the papillæ. The throat was still sore; the soft palate and fauces were vividly injected, and the follicles enlarged; both tonsils were large and red, but free from ulceration or membrane. An effervescing citrate of potash draught was prescribed. In the evening the temperature rose to 103·2°. The patient did not sleep well, and next morning (April 15th) she still complained of pain and dryness of the throat; she haa frequent sneezing, and the eyes were still watery. The temprature was 101·4°, and pulse 128. No urine had been passed since admission.—The appearance of the eruption was quite altered. Over the hands and arms it formed a continuous bright redness, like the eruption of scarlet fever; but over the front of the chest it had still a mottled character, although the patches ran into one another much more thon on admission. On April 16th, or the fifth day of the attack, the patient was much better.—The pulse was 100, and temperature 99°. She had slept well and had no cough, sneezing, running from the eyes, or sore-throat. The tongue was clean and red, and the appetite was returning. Urine abundant, alkaline, 1030; no albumen.—The eruption had almost disappeared from the face, but was still abundant and more confluent on the trunk and extremities. On April 17th all sign of fever was gone, but traces of the eruption were visible till the 19th. During convalescence there was abundant branny desquamation, but no albumen in the urine. On April 30th the patient left the hospital.

On April 14th I was called to see a young lady residing in the same house as Elizabeth R——, who had been taken on the same day with similar symptoms, followed next morning by an eruption all over the body, which at the time of my visit presented precisely the same characters as those above described. This child's attack was milder than even that of Elizabeth R——. The fever was very slight; and on April 16th the eruption had quite left the face, but was still visible on the legs. About ten or fourteen days subsequently, a second child in the same family had a similar attack.

The ailment from which these patients suffered is not generally recognised as a distinct disease; and cases of it, when they occur, are apt to be puzzling, and sometimes to get the medical attendant into trouble from his inability to determine their real nature. Yet, on the whole, they are not very rare. To explain the pathological relations of the disease, and also the reason of its being called "German measles," it is necessary to depart from the usual custom of a clinical lecture, and go into a little detail respecting the early history of measles and scarlet fever.

Measles and scarlet fever were long regarded as varieties of small-pox. Measles was first distinguished from variola by Abu Dschafar and other Arabian physicians in the 12th century; but measles and scarlet fever continued to be looked upon as one disease, which was designated "morbilli." An Italian physician, Phillip Ingrassias, of Palermo, in the middle of the sixteenth century, first described scarlet fever, which he called "rosalia," as distinct from the morbilli or measles. He pointed out that the rash of the former differed from that of the latter in being attended by little tumefaction, and in being diffused like that of erysipelas, the whole skin looking as if it were on fire. He adds: "Nonnulli sunt qui *morbillos* idem cum *rossalia* existimant; nos autem sœpe distinctos esse affectus, nostrismet oculis, non aliorum duntaxat relationi confidentes, inspeximus." The term "scarlatina" is said to have been the vernacular name for the disease on the shores of the Levant, and was first adopted in a medical work by Prosper Martianus, another Italian physician, who, about the middle of the sixteenth century, also described the disease as distinct from morbilli. Epidemics of scarlet fever were first described in this country by Sydenham, in 1676, and about the same time in Scotland, by Sir Robert Sibbald, physician to Charles II., and in the middle of last century by Fothergill and Huxham. But notwithstanding the accurate descriptions of these distinguished observers, scarlet fever and measles continued to be regarded by many physicians as mere varieties of one disease, the former being often styled "morbilli confluentes;" and the matter was only finally set at rest by Dr. William Withering in his classical essay published in 1779.*

---

*An Account of the Scarlet Fever and Sore-throat. London, 1779.

Shortly before, (1768) the two diseases had been separated by Sauvages in his Nosology, and he was the first to call measles "rubeola," instead of "morbilli," by which name it had always been known before. This new name, "rubeola," was adopted by Cullen in his Nosology, published four years later, (1772.)

The disease which I wish at present to bring under your notice was separated from measles and scarlet fever at a still later date. It was first described by German physicians about the end of the last and beginning of the present century, and particularly by Ziegler, Heim,* and Hildenbrand.† The last of these writers called the new disease "rubeola," and retained the name "morbilli" for measles proper; and this nomenclature has been adopted by many subsequent German writers, including Schönlein; whereas English writers, with the exception of Dr. Copland, have followed Cullen's nosology, and called ordinary measles "rubeola." Hence the rubeola of many German writers is not the rubeola of English nosologists, and when the new disease came to be recognized in England it was often designated "German rubeola or measles." There are, however, many other names by which it is known—such as rötheln, feüermasern, scarlatina morbillosa, morbilli scarlatinosi, rubeola notha, bastard measles or scarlatina, hybrid measles or scarlatina, &c.

In this country the disease has been well described by Dr. Robert Paterson, who observed it epidemic in Leith and its neighborhood in 1840;‡ and by Dr. G. W. Balfour, who in 1857 had an opportunity of studying another epidemic of it in the vicinity of Edinburgh.§

The existence of the disease, however, is still far from being generally recognized. With the exception of Copland and Aitken, few systematic writers in this country even refer to it. There is no allusion to it in Watson's classical lectures, nor in the new nomenclature of the Royal College of Physicians. Tanner mentions it, but thinks it unnecessary to describe it; while in Reynold's system of Medicine it is only alluded to as an error in diagnosis. Its existence as an independent disease is also doubted by many foreign physicians. Niemeyer, in his Text-book of Practical Medicine,‖ speaks of cases of scarlet fever with a rash like measles, (rubeola scar-

---

*Heim in Hufeland's Journal, 1812.

†Hildenbrand, Inst. Pract. Med., vol. iv., 412.

‡R. Paterson: An account of the Rotheln of German Authors, with a few observations on the Disease as it has been seen to prevail in Leith and its neighborhood. (Edin. Med. and Surg. Journ., Ap. 1840, p. 381.

§G. W. Balfour: Notice of an Epidemic of Rotheln—Rubeola? (Edin. Med. Journ., 1856–57, p. 717.)

‖Vol. ii., p. 543, American Transl.

latinosa) and of measles with a rash like scarlet fever (rubeola morbillosa,) but regards them as mere modifications of measles or scarlet fever. Hebra also, in his great work on Diseases of the Skin, refuses to admit the specific distinctness of rötheln.* The result is that few practitioners are acquainted with the disease, and many have never heard of it; and it is usually treated as a variety of measles or scarlet fever, although every now and then a medical man of more than usual discernment describes it in the journals as a new or anomalous exanthem.

But, whatever view we take of its pathology, the characters of the disease are sufficiently explicit, and deserve to be generally known. They may be briefly enumerated under the following heads:

1. *Premonitory fever*, with pains in the limbs and sometimes in the back; sore throat, with redness and swelling of the tonsils and fauces, coryza, sneezing and catarrh of the respiratory passages. In all cases there is sore throat; but the catarrh may be slight, or sometimes absent. Occasionally there is vomiting. Most authors fix the duration of this stage at about three days, the eruption being said to appear on the third or fourth day; but in my experience its duration, as in the case you have seen in the wards, has been sometimes much shorter, the rash appearing on the second day, or even within the first twenty-four hours.

2. *The rash* appears first on the breast and arms, but sometimes first on the face, and soon becomes universal. It consists of red elevated stigmata or dots, which run together into irregular patches, with obtuse blunt angles, something like those of measles; but, after a time, these patches usually coalesce, and the whole skin becomes uniformly red, as in scarlet fever. The eruption is copious, in a direct ratio to the severity of the general symptoms. It lasts longer, as a rule, than the rash of either measles or scarlet fever—from four to ten days. Its disappearance is followed by a desquamation of branny scales.

3. With the appearance of the rash the other symptoms are aggravated, and there is a *combination of scarlatinous angina and tongue with morbillous catarrh.* The throat is always sore, and the tonsils swollen and red; but the latter are rarely ulcerated. The swelling in the throat may be so great that the patient is unable to swallow; and occasionally, but not often, the glands in the neck suppurate. The tongue, which at first is white and coated, usually becomes, after a few days, clean and red, and the papillæ may be large and prominent, exactly as in

*Syd. Soc. Ed., vol. i., pp. 166, 167, 299.

the tongue of scarlet fever. But with all this there is more or less catarrh of the nasal and respiratory passages and coryza, and sometimes there is severe bronchitis, the suffering from which is greatly aggravated by the swelling in the throat. It is a mistake, therefore, to speak of rotheln as identical with what has been called "rubeola sine catarrho."

*The disease can propagate itself.* Many writers, like Copland, regard rotheln as a hybrid between measles and scarlet fever; and there are several circumstances which lend weight to this view—such as the fact that the disease presents the characters of both measles and scarlet fever combined, those of measles in one case, or of scarlet fever in another, or at different periods in the same case, being the more prominent; so that the two diseases are sometimes believed to occur simultaneously in the same house, or the one is thought to pass into the other in the same individual; or, again, the circumstance that rotheln has often been observed when measles and scarlet fever have both been epidemic. It is not correct, however, to say, as Hebra does, that rotheln occurs only in the sporadic form. Epidemics of it have been observed in Germany and Scotland.* But a curious and important fact is that, when the disease spreads, it does not propagate either measles or scarlet fever, as a hybrid of these two diseases might be expected to do, but a disease like itself. Of this I have had good evidence on several occasions; and the cases which I have brought under your notice furnish an additional illustration of the fact.

5. It has been a common observation by those who have paid attention to the subject that *rocheln does not protect from either measles or scarlet fever, and that a previous attack of either of these diseases does not protect from it.*† Both of the patients whose cases I have described to you had previously passed through attacks of scarlet fever and measles.

From these considerations it is obvious that rotheln, although partaking of the characters of both measles and scarlet fever, has some claim to be reckoned specifically distinct from both.

*Prognosis.*—The disease is in most instances mild, and a much more favorable prognosis may be formed than in true scarlet fever. Occasionally, however, the disease is severe or fatal, and in rare instances it is followed by dropsy.

The only *treatment* required in most cases is that the patient should remain in bed, and take a mild aperient, followed by a saline diaphoretic mixture. Occasionally the guttural or catarrhal symptoms will require special treatment.

---

*R. Patterson, loc. cit., p. 386.

†G. W. Balfour, loc. cit., p. 719; R. Paterson, loc, cit., p. 387; also, Ed. Med. Journ., 1855-6, p. 1133.

